# Potential metabolic resistance mechanisms to ivermectin in *Anopheles gambiae*: a synergist bioassay study

**DOI:** 10.1186/s13071-021-04675-9

**Published:** 2021-03-20

**Authors:** Patricia Nicolas, Caroline Kiuru, Martin G. Wagah, Martha Muturi, Urs Duthaler, Felix Hammann, Marta Maia, Carlos Chaccour

**Affiliations:** 1 ISGlobal, Hospital Clínic-Universitat de Barcelona, Rosello 132, 5ª 2ª, 08036 Barcelona, Spain; 2 Centro de Investigação em Saúde de Manhiça, 1929 Maputo, Mozambique; 3 Wellcome Sanger Institute, Wellcome Genome Campus, Hinxton, Cambridge, CB10 91SA UK; 4 Department of Biosciences, KEMRI Wellcome Trust Research Programme, Kilifi, 230-80108 Kenya; 5 Division of Clinical Pharmacology and Toxicology, Department of Biomedicine, University and University Hospital Basel, 4056 Basel, Switzerland; 6 Division of Clinical Pharmacology and Toxicology, Department of Pharmaceutical Sciences, University of Basel, 4056 Basel, Switzerland; 7 Division of Clinical Pharmacology and Toxicology, Department of Internal Medicine, University Hospital Bern, 3010 Bern, Switzerland; 8 Nuffield Department of Medicine, Centre for Tropical Medicine and Global Health, University of Oxford, Oxford, UK; 9 Ifakara Health Institute, Ifakara, 67501 United Republic of Tanzania; 10 Facultad de Medicina, Universidad de Navarra, 31008 Pamplona, Spain

**Keywords:** Ivermectin, Endectocide, Resistance, Insecticide resistance, CYP, P-gp, ABC transporter, Synergists, Bioassay

## Abstract

**Background:**

Despite remarkable success obtained with current malaria vector control strategies in the last 15 years, additional innovative measures will be needed to achieve the ambitious goals for malaria control set for 2030 by the World Health Organization (WHO). New tools will need to address insecticide resistance and residual transmission as key challenges. Endectocides such as ivermectin are drugs that kill mosquitoes which feed on treated subjects. Mass administration of ivermectin can effectively target outdoor and early biting vectors, complementing the still effective conventional tools. Although this approach has garnered attention, development of ivermectin resistance is a potential pitfall. Herein, we evaluate the potential role of xenobiotic pumps and cytochrome P450 enzymes in protecting mosquitoes against ivermectin by active efflux and metabolic detoxification, respectively.

**Methods:**

We determined the lethal concentration 50 for ivermectin in colonized *Anopheles gambiae*; then we used chemical inhibitors and inducers of xenobiotic pumps and cytochrome P450 enzymes in combination with ivermectin to probe the mechanism of ivermectin detoxification.

**Results:**

Dual inhibition of xenobiotic pumps and cytochromes was found to have a synergistic effect with ivermectin, greatly increasing mosquito mortality. Inhibition of xenobiotic pumps alone had no effect on ivermectin-induced mortality. Induction of xenobiotic pumps and cytochromes may confer partial protection from ivermectin.

**Conclusion:**

There is a clear pathway for development of ivermectin resistance in malaria vectors. Detoxification mechanisms mediated by cytochrome P450 enzymes are more important than xenobiotic pumps in protecting mosquitoes against ivermectin.
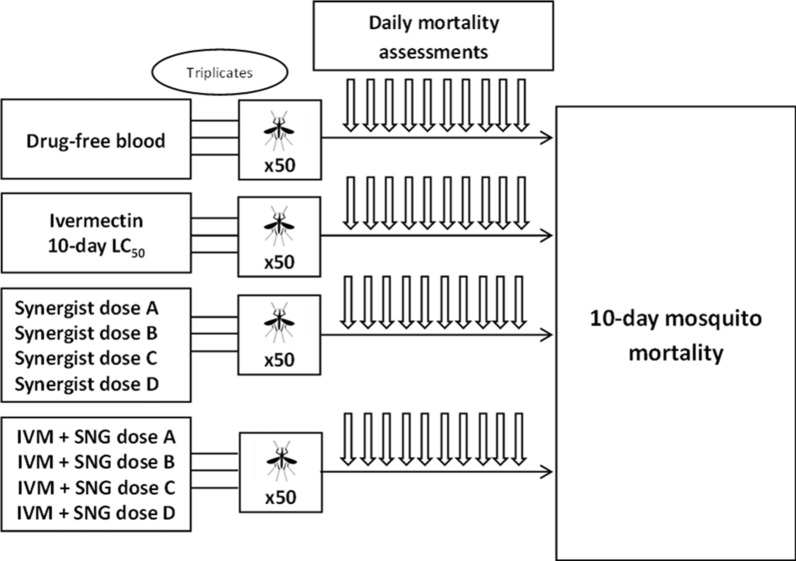

## Background

Since the turn of the century, significant advances have been made against malaria; the global malaria mortality rate has reduced by more than 50%, saving more than 6.8 million lives [1, 2]. Two vector control measures are mainly responsible for this success, (1) the use of insecticide-treated nets and (2) indoor residual spraying, both of which are insecticide-based and home-centered [3]. The continuous use and reliance on insecticides has put selective pressure on the mosquitoes, radically changing the vector species’ distribution and behavior [4]. This allows malaria transmission to continue by shifting to times and spaces unprotected by the current vector control measures, most noticeably early biting and/or outdoor biting [5]. Moreover, the selection pressure has yielded mosquito populations that are resistant to the current insecticides used in malaria vector control [6].

The new challenges in vector control urgently call for the development of new tools to circumvent them [7]. One of the proposed tools currently under evaluation is the use of endectocides [8]. Endectocides are antiparasitic drugs with activity against endoparasites and ectoparasites such as mosquitoes which feed on treated humans or animals. Importantly, endectocides can target exophilic and exophagic vectors, tackling the problem of residual transmission, while perfectly complementing the indoor vector control measures [9].

Owing to its excellent safety profile and activity against most malaria vectors, ivermectin is the leading endectocide candidate for malaria control [10, 11]. In addition, ivermectin is used extensively for the control of neglected tropical diseases (NTDs) that overlap with malaria in endemic areas, potentially increasing the cost-effectiveness of implementing ivermectin mass drug administration (MDA) [12, 13].

Several of the effects ivermectin has on malaria vectors point towards a low risk and slow speed for the development of resistance. These include (1) its mechanism of action, agonizing glutamate-gated chloride channels, which differs from all currently approved public health insecticides [14]; (2) direct delivery to the midgut with a blood meal that can bypass resistance associated with cuticular mechanisms [15]; and (3) marked reduction in fertility and fecundity of malaria vectors exposed to sublethal concentrations [16, 17]. Although the development of ivermectin resistance in malaria vectors may take a long time, it is inevitable, as it has already been reported in other arthropods [18]. Moreover, considering that ivermectin MDA for NTDs has been ongoing for the past 30 years, these could have potentially exposed malaria vectors to mostly sublethal ivermectin concentrations that could enhance the process of resistance development [19]. Therefore, it is important to have an early and thorough understanding of potential mosquito detoxification mechanisms for ivermectin. This will be crucial for early development of approaches that could delay, counter, or detect ivermectin resistance.

Herein, we evaluated the potential detoxification mechanisms involved in the response to ivermectin in the *Anopheles gambiae* s.s. mosquito. We were mainly interested in two general mechanisms of detoxification: (1) metabolic detoxification mediated by enzymes, specifically cytochrome P450 enzymes (CYPs), and (2) detoxification by excretion facilitated by ATP binding cassette (ABC) transporters, specifically P-glycoprotein (P-gp). The increased activity of CYPs leads to increased biodegradation of the toxins, while increased activity of P-gp leads to increased excretion of xenobiotics. Both mechanisms reduce the toxic effects of the compounds by decreasing the insects’ systemic exposure to them [20]. Both detoxification mechanisms have been implicated in resistance to insecticides used for the control of malaria vectors, with metabolic detoxification being the most common mechanism [21, 22]. Elevated levels of CYPs, esterases, and glutathione S-transferases (GSTs) are associated with resistance to different classes of insecticides [22]. Though both mechanisms are known to contribute to ivermectin resistance in other arthropods, their potential contribution to ivermectin resistance in mosquitoes has not been thoroughly explored [23–25].

In this study, we assessed the interaction of different chemical inhibitors and inducers of CYP and P-gp with ivermectin in *An. gambiae* s.s. (Kilifi strain) mosquitoes. Our main questions were whether and how the inhibitors and inducers of CYP and P-gp affect ivermectin-induced mosquito mortality.

## Methods

### Experimental design

**Fig. 1:**
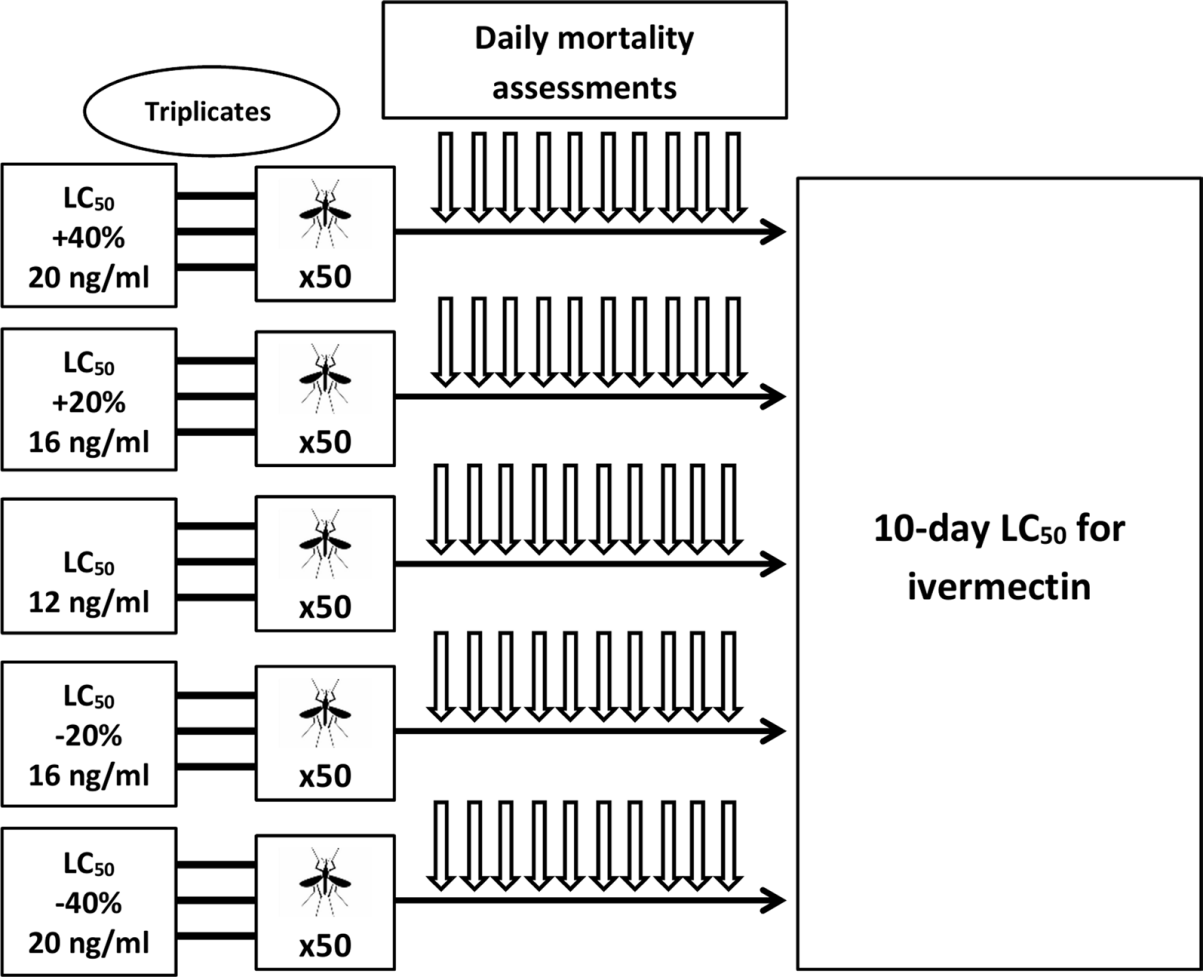
Schematic representation of phase 1 experiments. Dose-finding study for ivermectin’s 10-day insecticidal concentration LC_50_ in our *Anopheles gambiae* s.s. colony. LC_50_: insecticidal concentration 50

The experiments were conducted in two phases. In phase 1, an ivermectin dose-finding experiment was performed using triplicate batches of 50 female mosquitoes (3–5 days old) with the aim of identifying the 10-day LC_50_ of ivermectin for our colony (Fig. 1). The 10-day period was chosen based on the minimum extrinsic incubation period of *Plasmodium falciparum* parasites, i.e. mosquitoes dying before 10 days are unlikely to become infectious [26]. We tested five different concentrations of ivermectin spanning ± 20–40% of the 5-day LC_50_ described by Kobylinski et al. [27], namely 4 ng/ml, 8 ng/ml, 12 ng/ml, 16 ng/ml, and 20 ng/ml.

**Fig. 2:**
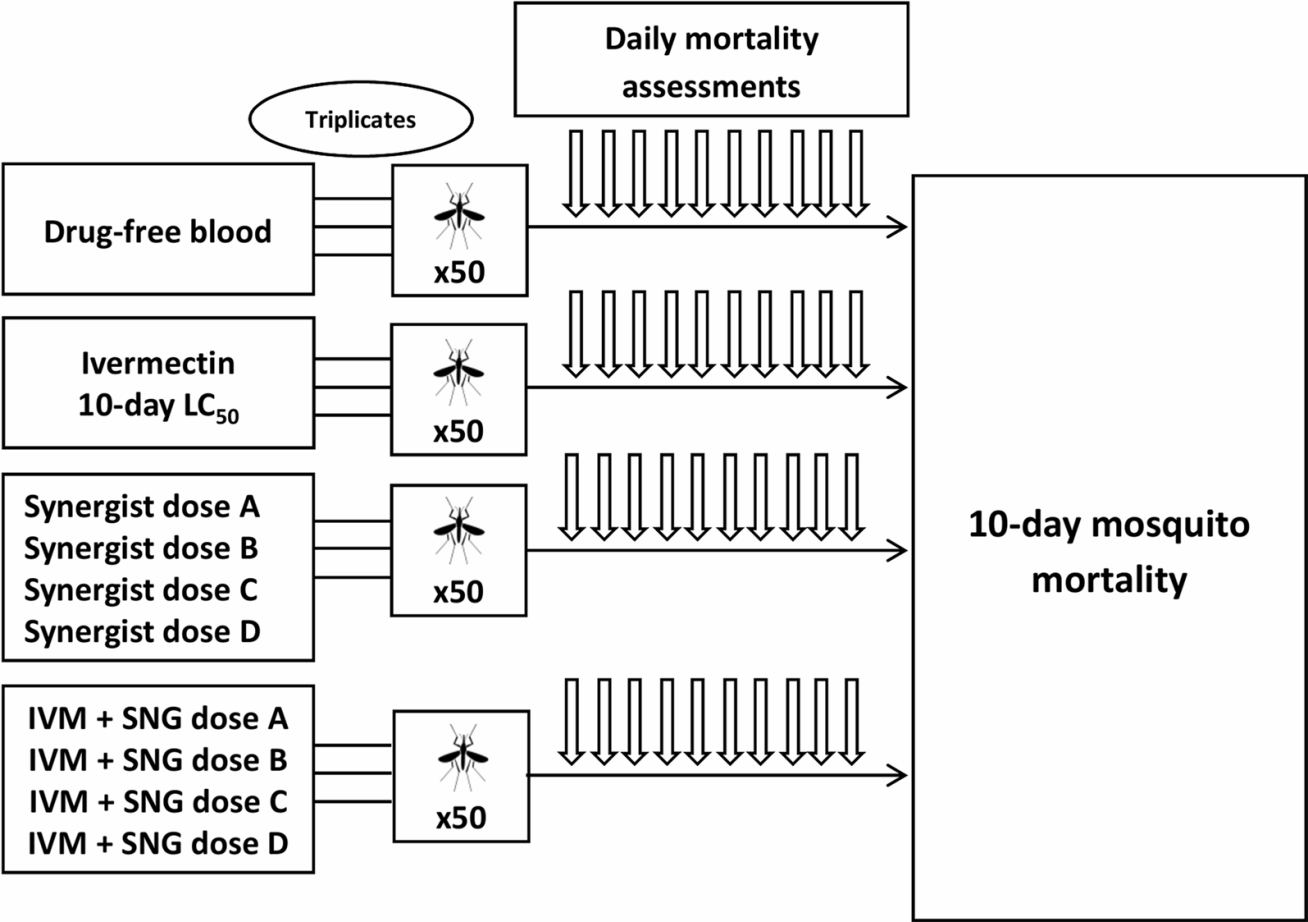
Schematic representation of phase 2 experiments. Assessment of 10-day mosquito mortality after feeding on blood containing different concentrations of CYP/P-gp inhibitors and inducers, either alone or in combination with ivermectin at the 10-day LC_50_ dose determined in phase 1

In phase 2, following the identification of a concentration of ivermectin yielding about 50% mortality in 10 days, we evaluated (1) the effect on mosquito mortality of CYP and/or P-gp inhibitors and inducers alone at different concentrations and (2) the effect of combining ivermectin with different doses of CYP and/or P-gp inhibitors and inducers (Fig. 2).

For voriconazole, ritonavir, cobicistat, cyclosporine A, elacridar, and rifampicin, the concentrations tested were based on the maximum blood concentration reached in humans after a single dose (*C*_max_) as reported in the literature (Table 1). Four concentrations corresponding to *C*_max_, 75% *C*_max_, 50% *C*_max_ and 25% *C*_max_ were evaluated. Hereafter, *C*_max_ concentration is referred to as A, 75% *C*_max_ as B, 50% *C*_max_ as C, and 25% *C*_max_ as D.

**Table 1:** Synergists and corresponding doses used for the experiments

Drug	Mechanism of action	*D*_A_ (*C*_max_) (ng/ml)	*D*_B_ (75% of *C*_max_) (ng/ml)	D_C_ (50% of *C*_max_) (ng/ml)	*D*_D_ (25% of *C*_max_) (ng/ml)
Cobicistat [31]	Dual CYP/P-gp inhibitor^a^	990	742	495	247
Cyclosporine A [32]	Selective P-gp inhibitor^b^	1802	1351	901	450
Elacridar [33]	Selective P-gp inhibitor	160	120	80	40
Rifampicin [34]	Dual CYP/P-gp inducer	7000	5250	3500	1750
Ritonavir [35]	Dual CYP/P-gp inhibitor	11,000	8250	5500	2750
Voriconazole [36]	Dual CYP/P-gp inhibitor	3667	2750	1833	916

^a^With effect markedly skewed towards CYP inhibition

^b^Negligible effect on CYPs

Given that mosquitoes were to be exposed in batches to the different drugs and combinations, the study was considered cluster-randomized, in which the batch exposed to any drug was the unit of randomization and the mosquito was the analysis unit. The sample size was adjusted for a cluster effect. A 50% increase in ivermectin-driven 10-day mosquito mortality was considered of potential public health value. According to the method reported by Hayes and Bennett [28], using three replicas of 50 mosquitoes per group gives the study 80% power at a 5% significance level to detect a 50% increase in 10-day mortality from 50 to 75% by adding the synergist. This uses an intra-cluster correlation coefficient of 0.06 described before for mosquito colonies [29]. These calculations are confirmed using the formula of Gangnon and Kosorok [30], which shows a design effect of 3.34, with 70% possibility of observing mortality within 10 days.

### Mosquitoes

Throughout the study we used an *Anopheles gambiae* s.s. Kilifi strain maintained in KEMRI–Wellcome Trust Research Programme insectary in Kilifi, Kenya. The colony was adapted to the insectary in 2011 from larvae collected in Mbogolo, Kilifi county. The colony is fully insecticide-susceptible and is subjected to quarterly resistance monitoring using WHO tube tests.

The mosquitoes were maintained at 28 °C and 80% relative humidity at a 12-h light:12-h dark photoperiod. Adult mosquitoes were fed ad libitum 10% glucose solution via impregnated cotton wool, while larvae were fed TetraMin fish flakes.

### Experimental drugs

Based on their mechanism of action in humans, voriconazole, ritonavir, and cobicistat were classified as dual CYP/P-gp inhibitors [18, 19], cyclosporine A and elacridar were classified as P-gp-specific inhibitors [37], and rifampicin was classified as a dual CYP/P-gp inducer [38]. The choice of inhibitors and inducers used in the present study was based on their ability to act as substrates for the CYP CYP3A4, which is the major enzyme involved in ivermectin metabolism in humans [39].

Ivermectin, voriconazole, ritonavir, cobicistat, cyclosporine A, elacridar, and rifampicin were obtained from Sigma Aldrich (Spain). The active pharmacological ingredients were dissolved in dimethyl sulfoxide (DMSO) to prepare stock solutions for all compounds. Aliquots of the prepared solutions were frozen at −20 °C. For each experiment, the stock solutions were diluted in phosphate-buffered saline (PBS) to achieve the desired concentration.

### Membrane blood feeding

For blood feeding, we used certified drug-free cattle blood defibrinated using the Reynold’s method [40]. Briefly, after blood collection, the blood was gently shaken for 5–10 min in a 250 ml glass bottle containing a copper wire arranged as an elongated spiral with two outer loops inside. The wire was then removed together with the fibrin that became bound to it [40]. The defibrinated blood was mixed with the drugs at appropriate concentrations to a total volume of 6 ml. In the case of the control group, the blood was mixed with PBS only.

Fifty 2–5-day-old blood-naïve female mosquitoes were transferred from stock cages to mosquito holding cups (1000 cm^3^) and starved of water and glucose for 6–8 h before blood feeding. The holding cups were covered by untreated net with a lateral aspiration hole covered with a double layer of dental dam. Blood feeding was done using an inverted cup technique [41]. The blood was placed on the bottom surface of a paper cup (500 ml capacity) and covered by a thinly stretched Parafilm membrane. The membrane was secured with masking tape, and the cup was inverted and filled with warm water (~ 38 °C). The cups were then held over the netting material of the holding cups and mosquitoes were allowed to feed. Mosquito feeding was done in the dark for a period of 30–60 min. Visually unfed mosquitoes were removed and only fully engorged mosquitoes kept in the holding cages for follow-up and maintained at standard insectary conditions. Mortality was monitored every 24 h for 10 days by counting and removing dead mosquitoes. At least three replicates were performed for every drug or drug combination tested. The position of the cages was rotated daily.

### Data collection and statistical analysis

Daily mortality data were entered in Excel spreadsheets. Survival Kaplan–Meier and Cox regression analyses were performed in Addinsoft XLSTAT^®^ version 2018.5 software (New York, NY, USA) and GNU R (R Core Team [2020] R: A language and environment for statistical computing, version 3.6.3, R Foundation for Statistical Computing, Vienna, Austria, https://www.R-project.org). Comparisons of survival patterns were performed with the log-rank test using a 5% significance level. When the overall *p* value was < 0.05, pairwise comparisons were performed and Bonferroni correction used to correct for multiple comparisons.

## Results

### Ivermectin induces dose-dependent but delayed mortality in *An. gambiae*

**Fig. 3:**
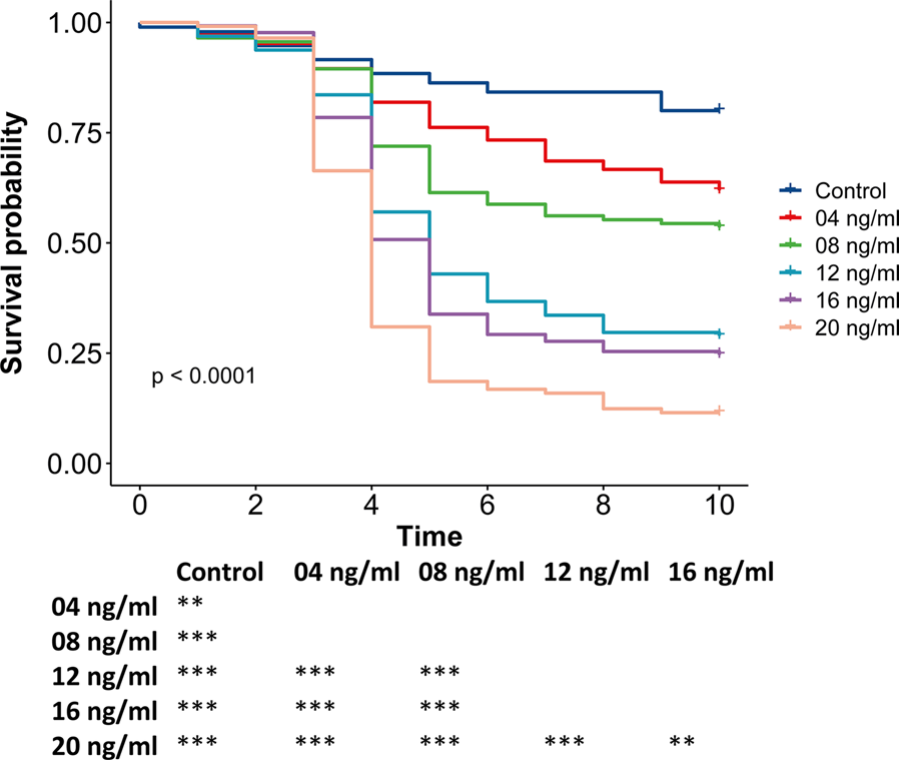
Ivermectin induces dose-dependent but delayed mortality in *An. gambiae*. Daily survival probability for *An. gambiae* mosquitoes after ingesting blood containing different concentrations of ivermectin. Table shows pairwise comparisons of survival in different concentrations, with significance level indicated: **p < 0.01, ***p < 0.001. The 10-day LC_50_ is between 8 and 12 ng/ml for this strain in this insectary

We first determined the 10-day LC_50_ of ivermectin in our colony of *An. gambiae* s.s. The effect of ivermectin on mosquito survival was dose-dependent (Fig. 3). The 8 ng/ml concentration was the only one that resulted in approximately 50% mortality in 10 days, showing a mortality rate of 46.49% (Table 2). Therefore, this concentration of ivermectin was chosen for use in phase 2 experiments.

**Table 2:** Mosquito survival after ingestion of multiple concentrations of ivermectin

Ivermectin concentration (ng/ml)	Mean survival time (95% CI)	10-day % mortality
0 (control)	8.1 (7.6–8.5)	20
4	8.1 (7.5–8.6)	38.1
8	7.3 (6.8–7.9)	46.5
12	6.0 (5.5–6.5)	71.1
16	5.6 (5.2–6.1)	75.4
20	4.5 (4.1–4.9)	88.5

Mortality after ivermectin intake did not occur immediately and was negligible for the first 3 days. Instead, mosquito mortality occurred largely between days 4 and 6. Most of the mosquitoes surviving after day 6 remained alive until day 10. These tendencies were observed across all the concentrations of ivermectin.

### Dual CYP/P-gp inhibitors have a synergistic effect on ivermectin-induced mosquito mortality

**Fig. 4:**
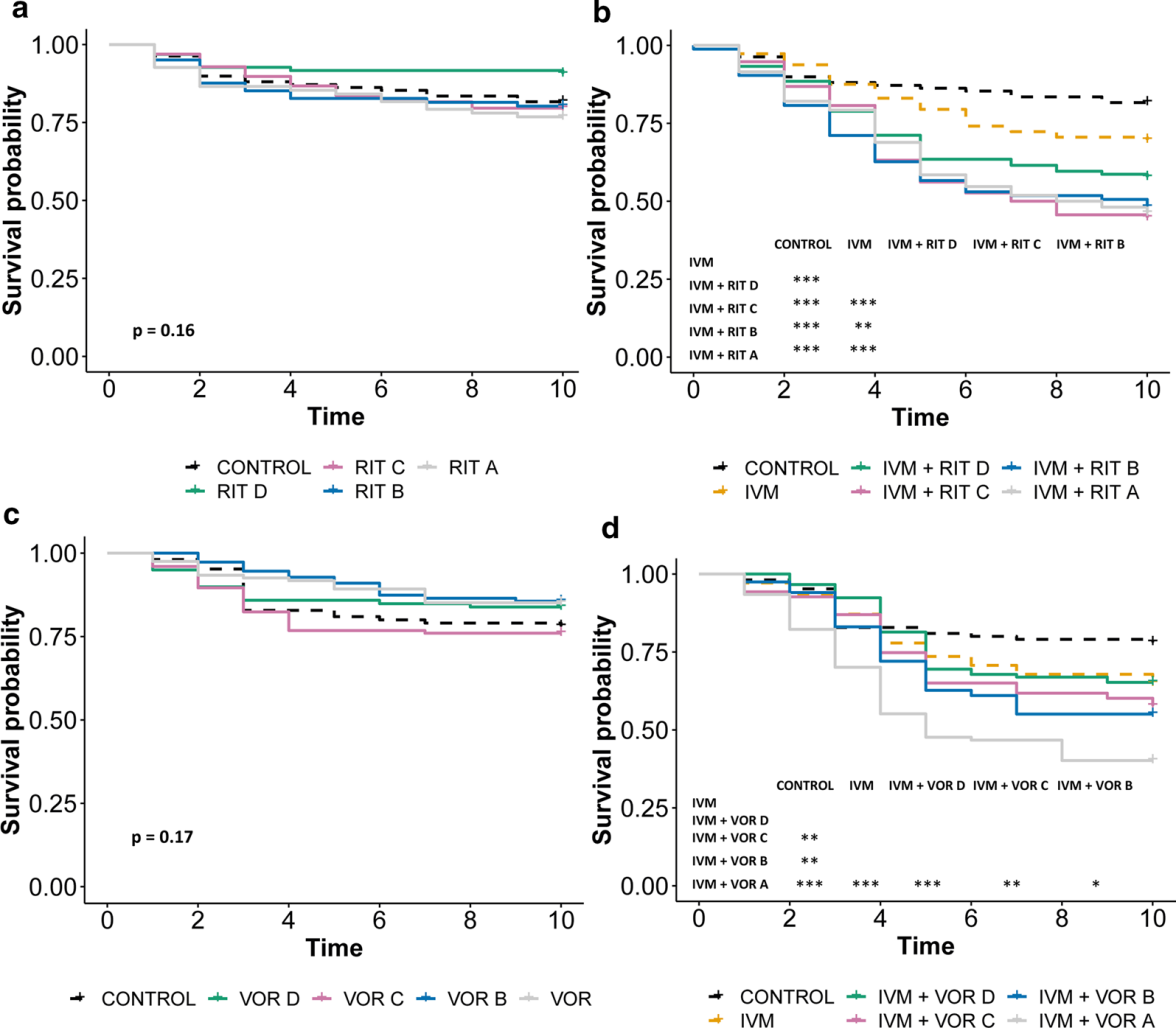
Synergistic effect of dual CYP/ P-gp inhibitors on ivermectin-induced mosquito mortality. Daily survival probability of *An. gambiae* mosquitoes after imbibing blood containing (**a**) varied concentrations of ritonavir (RIT), (**b**) ivermectin (IVM) mixed with varied concentrations of ritonavir, (**c**) varied concentrations of voriconazole, and (**d**) ivermectin mixed with varied concentrations of voriconazole. When the overall *p* value was < 0.05, pairwise comparisons was performed and the significance level indicated: *p < 0.05, **p < 0.01, ***p < 0.001. Concentration A > B > C > D

**Fig. 5:**
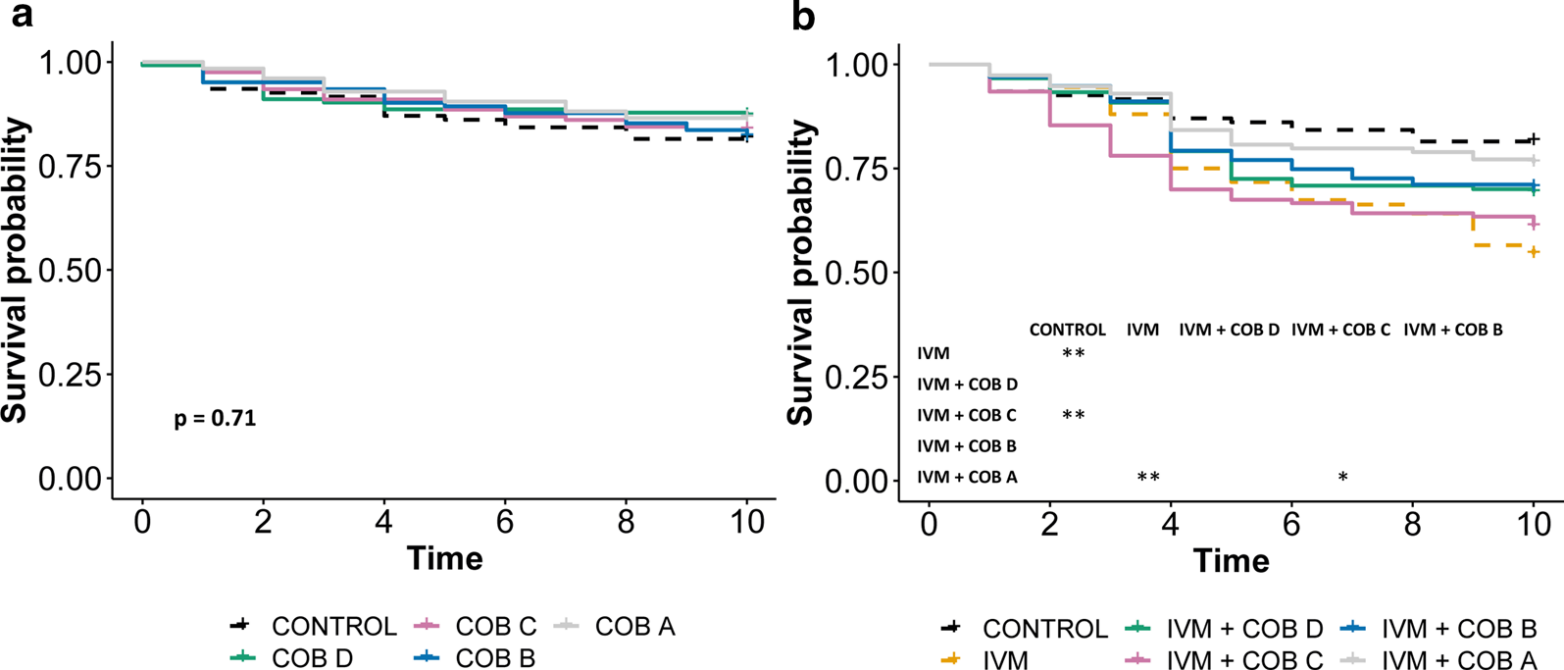
Cobicistat appears to antagonize the ivermectin-induced mosquito mortality. Daily survival probability of *An. Gambiae* mosquitoes after ingesting blood containing (**a**) varied concentrations of cobicistat (COB), and (**b**) ivermectin (IVM) mixed with varied concentrations of cobicistat. When the overall *p* value was < 0.05, pairwise comparisons were performed and the significance level indicated: *p < 0.05, **p < 0.01, ***p < 0.001. Concentration A > B > C > D

Firstly, we excluded the possibility that any observed differences in mortality observed by combining ivermectin with a CYP/P-gp inhibitor were because of additive mortality caused by the CYP/P-gp inhibitors themselves. No significant differences in mortality were observed between mosquitoes that consumed CYP/P-gp inhibitors alone and those that did not, irrespective of the dose used (Figs. 4a, c, 5a).

In contrast, differences in mortality were observed between mosquitoes that consumed ivermectin alone and those that consumed ivermectin combined with a dual CYP/P-gp inhibitor. Notably, experiments conducted with ritonavir and voriconazole showed enhanced ivermectin-induced mortality and dose-dependent synergism (Fig. 4b, d). Unlike voriconazole, the synergistic effect of ritonavir saturated at the second lowest concentration (concentration C). Additionally, when ivermectin was combined with ritonavir or voriconazole, an increase in the percentage of mortality and decrease in mean survival time of the mosquitoes were observed (Table 3).

**Table 3:** Synergist drugs combined with ivermectin

Drug	Synergist concentration (ng/ml)	Mean survival time (95% CI)	10-day % mortality
Voriconazole			
Control		8.5 (8.0–9.1)	21.9
Ivermectin		8.0 (7.5–8.5)	35.0
IVM + VOR A	3667	5.4 (4.9–5.9)	59.8
IVM + VOR B	2750	5.7 (5.3–6.0)	44.9
IVM + VOR C	1833	7.6 (7.0–8.1)	42.2
IVM + VOR D	916	7.4 (6.9–7.8)	34.7
Ritonavir			
Control		8.0 (7.5–8.4)	18.3
Ivermectin		8.2 (7.7–8.8)	30.3
IVM + RIF A	11,000	6.8 (6.1–7.5)	53.7
IVM + RIF B	8250	6.6 (5.8–7.4)	51.8
IVM + RIF C	5500	6.7 (6.1–7.3)	55.2
IVM + RIF D	2750	7.3 (6.7–8.0)	42.3
Cobicistat			
Control		7.1 (6.8–7.5)	18.5
Ivermectin		7.8 (7.1–8.4)	45.6
IVM + COB A	990	8.6 (8.1–9.1)	23.6
IVM + COB B	742	8.2 (7.8–8.7)	29.6
IVM + COB C	495	7.5 (6.9–8.1)	39.0
IVM + COB D	247	8.1 (7.6–8.6)	30.8
Elacridar			
Control		9.5 (9.3–9.8)	12.2
Ivermectin		7.1 (6.5–7.6)	54.6
IVM + ELA A	160	6.9 (6.4–7.4)	62.3
IVM + ELA B	120	7.3 (6.8–7.8)	49.6
IVM + ELA C	80	6.5 (6.1–7.0)	51.4
IVM + ELA D	40	7.0 (6.5–7.4)	56.1
Cyclosporine A			
Control		9.0 (8.3–9.6)	15.6
Ivermectin		9.0 (8.5–9.6)	21.8
IVM + CYC A	1802	7.8 (6.8–8.8)	34.7
IVM + CYC B	1351	7.7 (6.8–8.6)	38.1
IVM + CYC C	901	7.7 (7.0–8.4)	20.9
IVM + CYC D	450	8.5 (7.9–9.2)	33.3
Rifampicin			
Control		7.2 (6.6–7.7)	39.1
Ivermectin		6.7 (6.0–7.5)	53.4
IVM + RIF A	7000	6.9 (6.2–7.6)	49.5
IVM + RIF B	5250	6.4 (5.6–7.1)	61.3
IVM + RIF C	3500	8.2 (7.6–8.9)	31.5
IVM + RIF D	1750	6.4 (5.8–7–0)	66.3

*IVM* ivermectin, *VOR* voriconazole, *RIF* ritonavir, *COB* cobicistat, *ELA* elacridar, *CYC* cyclosporine A

### Cobicistat, a strong antagonistic effect

Cobicistat alone had no effect on mosquito mortality regardless of the concentration used (Fig. 5a). Despite being a dual CYP/P-gp inhibitor, cobicistat showed an antagonistic effect on ivermectin-induced mortality when combined with ivermectin. High cobicistat concentrations protected the mosquitoes from ivermectin toxicity, with a higher survival probability recorded in cobicistat combined with ivermectin than ivermectin alone (Fig. 5b). Additionally, when combined with ivermectin, cobicistat showed a lower percentage of mortality in comparison to ivermectin alone (Table 3).

### Inhibition of P-gp alone does not affect ivermectin-induced mosquito mortality

**Fig. 6:**
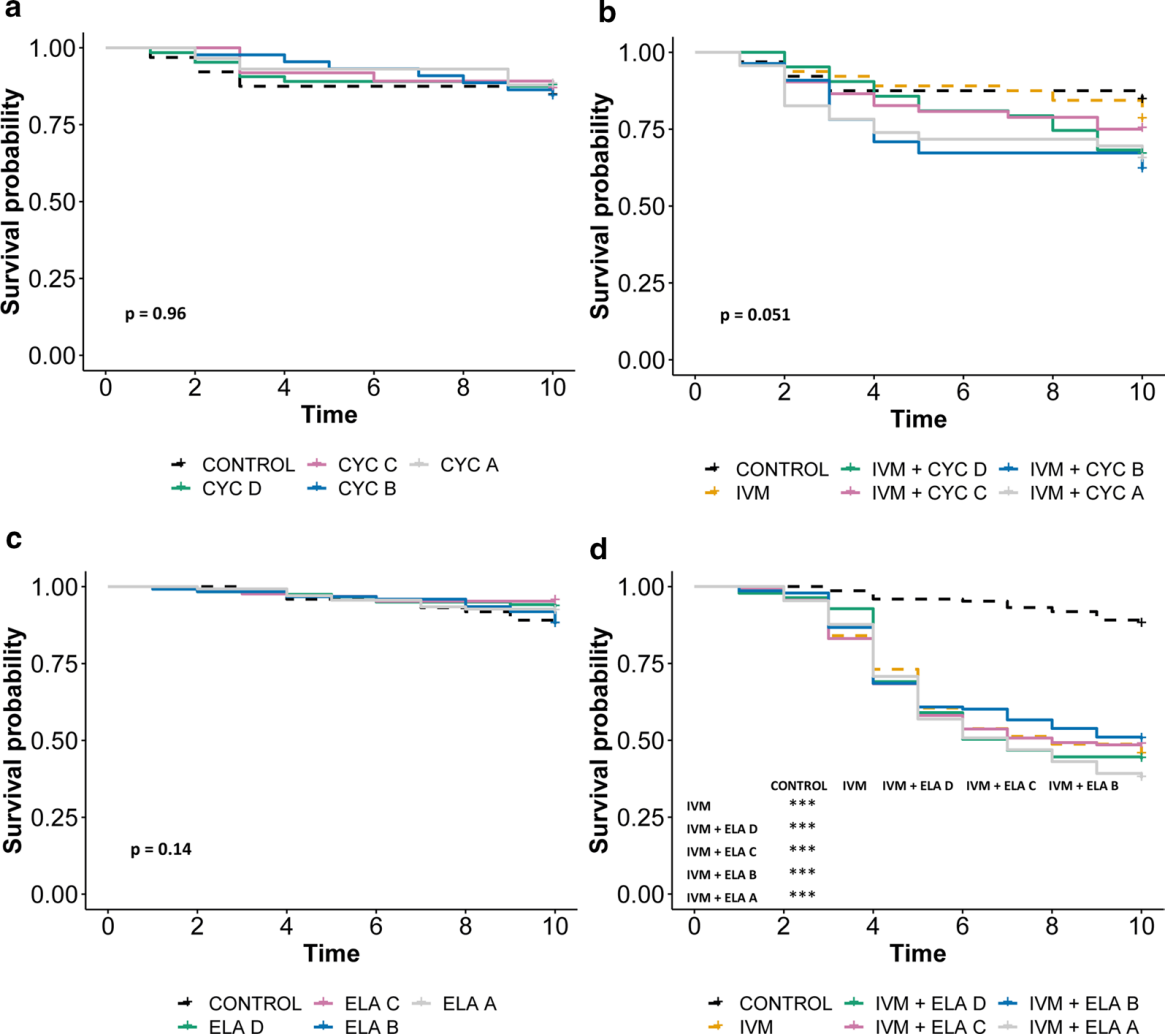
Inhibition of P-gp alone does not affect ivermectin-induced mosquito mortality. Daily survival probability of *An. Gambiae* mosquitoes after ingesting blood containing (**a**) varied concentrations of cyclosporine A (CYC), (**b**) ivermectin (IVM) mixed with varied concentrations of cyclosporine A, (**c**) varied concentrations of elacridar (ELA), and (**d**) ivermectin mixed with varied concentrations of elacridar. When the overall *p* value was < 0.05, pairwise comparisons were performed and the significance level indicated: *p < 0.05, **p < 0.01, ***p < 0.001. Concentration A > B > C > D

After observing the effect of CYP/P-gp inhibition on ivermectin-induced mortality, we next assessed the effects of only inhibiting P-gp transporters. For this, we used elacridar and cyclosporine A, which are predominantly P-gp inhibitors in vivo. Similarly to the dual CYP/P-gp inhibitors, the selective P-gp inhibitors alone did not cause significant mortality at any dose tested (Fig. 6a, c).

Moreover, when combined with ivermectin, both elacridar and cyclosporine A showed no effect on ivermectin-induced mortality, suggesting that P-gp inhibitors do not synergize with ivermectin to increase mortality (Fig. 6b, d).

### Simultaneous induction of cytochrome P450 and P-gp may confer modest protection from ivermectin-induced mortality

**Fig. 7:**
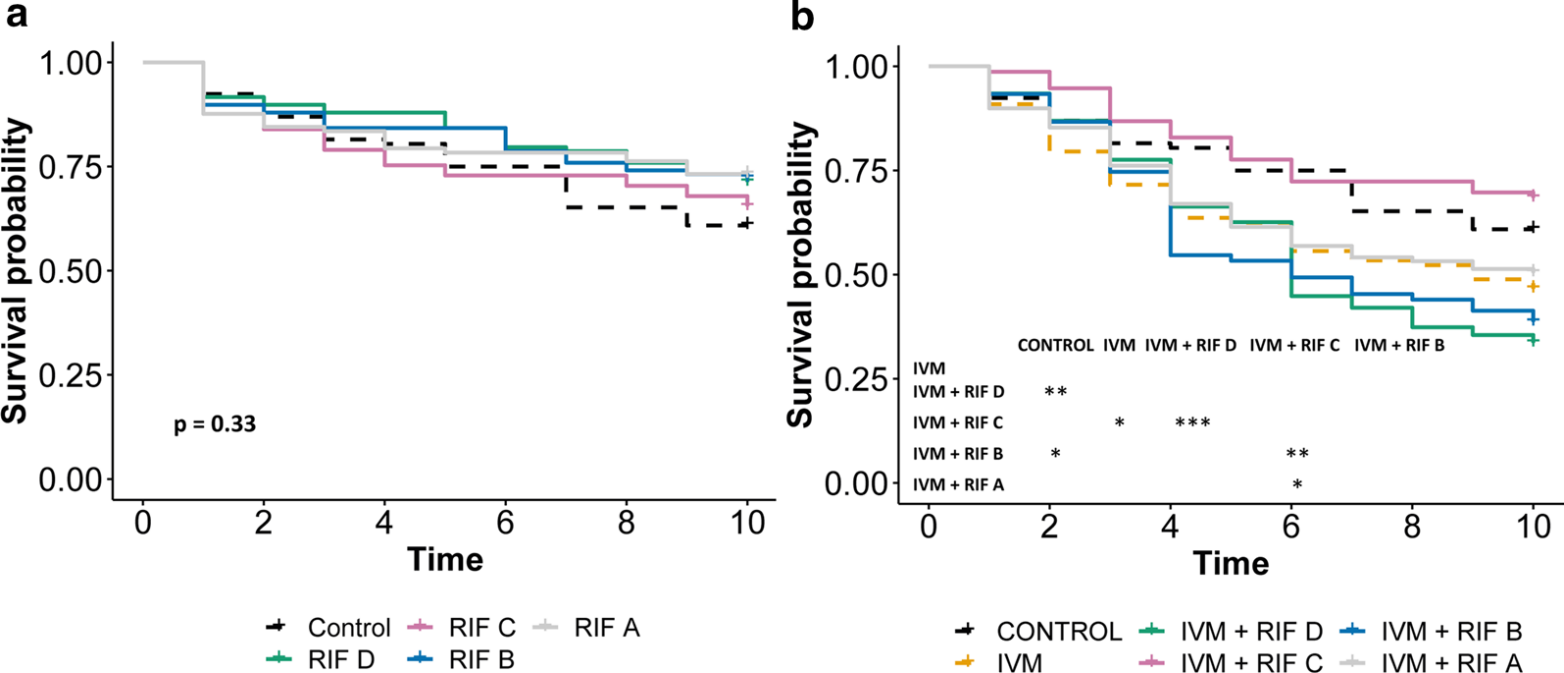
Simultaneous induction of cytochrome P450 and P-gp transporters may confer modest protection from ivermectin-induced mortality. Daily survival probability of *An. Gambiae* mosquitoes after ingesting blood containing (**a**) varied concentrations of rifampicin (RIF) and (**b**) ivermectin (IVM) mixed with varied concentrations of rifampicin. When the overall *p* value was < 0.05, pairwise comparisons were performed and the significance level indicated: *p < 0.05, **p < 0.01, ***p < 0.001. Concentration A > B > C > D

No significant difference was observed in the survival of mosquitoes feeding on rifampicin alone at the different doses tested (Fig. 7a).

However, when combined with ivermectin, rifampicin at a concentration close to 50% of its *C*_max_ for CYP 3A4 induction showed a statistically significant difference from ivermectin alone; this was seen as antagonism by reducing the ivermectin-induced mortality (Table 3; Fig. 7b).

## Discussion

Susceptibility to ivermectin has been shown to vary among mosquito species as well as among mosquito strains of the same species [27, 42, 43]. Depending on the time frame during which survival is monitored, *An. gambiae* have shown an LC_50_ of 19.8, 15.9, and 22.4 ng/ml when survival is monitored for 9, 7, and 5 days, respectively [27, 44, 45]. In our case, by monitoring survival for 10 days, we achieved 46.49% mortality with a concentration of 8 ng/ml. Though the levels of ivermectin in the blood drop rapidly, a concentration above 8 ng/ml can be maintained for close to 36 h following an ivermectin dose in humans [14, 27]. Our results are aligned with those of Smit et al., in which even very low ivermectin concentrations increased mosquito mortality if the follow-up period encompassed the usual lifespan [46].

At the doses used, ivermectin-induced mortality in mosquitoes was not observed until 2–3 days post-exposure. One potential explanation for this is the time taken for ivermectin to be absorbed from the midgut, as faster lethality onset has been observed when ivermectin is injected directly into the midgut than when it is taken as part of a blood meal [42]. The second plausible explanation for the delayed mortality is the involvement of ivermectin metabolites rather than the parent compound in causing mosquito mortality. There is accumulating evidence suggesting the involvement of ivermectin metabolites in mosquito mortality, though the specific metabolites are yet to be identified [46, 47]. However, even before the onset of lethality that is measurable with a 10-day follow up, ivermectin can potentially affect mosquito mortality in the wild due to its effects on locomotion [48]. Simultaneously, a reduction in locomotion ability would affect the vectorial capacity regardless of mosquito mortality, which could in turn further reduce malaria transmission.

Ivermectin-induced mortality is greatly dependent on attaining high systemic levels of ivermectin in the mosquito. The exposure to ivermectin is determined by the mosquito’s detoxification capacity. Generally, in insects, detoxification processes involve metabolic enzymes such as CYP, esterase, and glutathione-S-transferases (GSTs) together with efflux pumps like the P-gp [49]. In this study, we investigated whether and how CYPs and P-gp transporters affected ivermectin-induced mortality in mosquitoes. Our results demonstrate that the simultaneous inhibition of CYPs and P-gp transporters enhances ivermectin-dependent mortality in a dose-dependent manner, indicating synergism. However, this only happened selectively when ritonavir or voriconazole was used. Unexpectedly, the use of cobicistat, which is also a dual CYP/P-gp inhibitor, rendered some protection from ivermectin. Cobicistat is a structural analogue of ritonavir, but unlike ritonavir, which is known to inhibit and induce multiple CYPs, cobicistat more selectively inhibits CYP3A4 [50]. Though ritonavir and cobicistat are considered clinically equivalent, the small difference in ritonavir’s ability to induce CYPs could result in differences in the drug–drug interaction [51, 52]. The induction of CYPs could possibly lead to ivermectin metabolism, making ivermectin metabolites available.

Despite dual CYP/P-gp inhibitors showing an effect on ivermectin-induced mortality, P-gp selective inhibitors did not have a measurable effect. Taken together, our results suggest that detoxification mechanisms mediated by CYPs are more important in ivermectin detoxification. This is contrary to what has been reported in mosquito larval stages, where Buss et al. demonstrated that inhibition of P-gp using verapamil led to increased toxicity in *Culex* mosquitoes [23]. Collectively, both findings suggest heterogeneity in detoxification mechanisms in different stages of mosquito development. As a holometabolous insect, the changes between the immature stages (larvae and pupae) and the adult stage are characterized by differences in diet, habitat, morphology, physiology, and behavior. These differences could potentially lead to differences in evolution of protective mechanisms [53]. Larval stages are known to be more prone to developing insecticide resistance compared to the adult stage [53]. Whether the P-gp-mediated detoxification reported in *Culex* larvae is additive or alternative to CYP-mediated detoxification in larvae merits investigation. Larval habitats are often exposed to ivermectin through contamination of aquatic habitats with excreta from treated livestock. The stability of ivermectin in water for long periods increases the exposure of the larvae to ivermectin and could potentially accelerate the development of resistance [54]. It is important to understand the mechanisms behind larval resistance to ivermectin and whether they contribute to ivermectin resistance in adults.

Our results warrant the investigation of selective CYP inhibitors for the ability to synergize ivermectin-induced mortality, including molecules susceptible to administration by contact such as piperonyl butoxide (PBO). This will help answer the question of whether CYP inhibition independent of P-gp inhibition could still have practical implications. Notably, the current recommendation for tackling metabolic resistance to insecticides is the use of PBO [55]. PBO, which is an inhibitor of CYP450 enzymes and is currently in use by incorporation into pyrethroid long-lasting insecticidal nets (LLINs), could also enhance ivermectin-induced mortality. However, it must be first determined whether it synergizes ivermectin-induced mortality.

Limitations of this work include the following: (1) The ivermectin concentration used for the combined experiments did not quite yield 50% mortality in 10 days, but only 46%. (2) The initial solutions of synergistic molecules were prepared using small quantities of DMSO and then diluted in PBS to the desired concentration. The control solution did not contain DMSO, as previous experiments have shown that it does not alter mosquito survival at low concentrations [27]. (3) It is still unclear whether the finding that only one rifampicin concentration demonstrated an interaction with ivermectin is an artifact or an effect limited to a certain concentration range, and (4) it is also unclear why ritonavir and cobicistat interacted in such different ways with ivermectin when both molecules strongly inhibit CYP 3A4. Still, the involvement of CYPs in ivermectin metabolism could potentially lead to cross-resistance between ivermectin and current insecticides used in vector control. Deus et al. previously demonstrated that pyrethroid-resistant *Ae. aegypti* have higher tolerance to ivermectin [43]. This could potentially affect ivermectin susceptibility in mosquito populations already showing metabolic resistance to insecticides, and highlights the need to investigate the impact of insecticide resistance on susceptibility to ivermectin.

Nevertheless, ivermectin remains a good alternative for mosquito populations whose mode of resistance is via point mutations, since this resistance mechanism is not analogous between the current insecticides and ivermectin [56]. While resistance to insecticides is caused by mutations in the sodium channel, acetylcholinesterase, or gamma-aminobutyric acid (GABA) receptor genes, ivermectin resistance in other arthropods is associated with mutations involving the glutamate-gated chloride channels (GluCls) [57, 58].

In the case where ivermectin is to be used in mosquito populations with metabolic resistance to the current insecticides, our study provides insights into the possibility of ivermectin cross-resistance with other insecticides. Our results suggest that detoxification mechanisms mediated by CYPs are more important in ivermectin resistance than detoxification mediated by efflux pumps.

## Data Availability

All study data are contained within this manuscript.
